# Reduced Recovery Capacity After Major Trauma in the Elderly: Results of a Prospective Multicenter Registry-Based Cohort Study

**DOI:** 10.3390/jcm9082356

**Published:** 2020-07-23

**Authors:** Viola Freigang, Karolina Müller, Antonio Ernstberger, Marlene Kaltenstadler, Lisa Bode, Christian Pfeifer, Volker Alt, Florian Baumann

**Affiliations:** 1 Department of Trauma, Regensburg University Medical Center, 93053 Regensburg, Germany; antonio.ernstberger@ukr.de (A.E.); christian.pfeifer@ukr.de (C.P.); volker.alt@ukr.de (V.A.); florian.baumann@ukr.de (F.B.); 2 Center for Clinical Studies, Regensburg University Medical Center, 93053 Regensburg, Germany; karolina.mueller@ukr.de; 3 Department of Surgery, Regensburg University Medical Center, 93053 Regensburg, Germany; marlene.kaltenstadler@ukr.de; 4 Department of Orthopaedics and Trauma Surgery, Faculty of Medicine, Medical Center—Albert-Ludwigs-University of Freiburg, 79085 Freiburg im Breisgau, Germany; lisa.bode@uniklinik-freiburg.de

**Keywords:** health service research, management of major trauma, geriatric major trauma, outcome research, patient-reported outcome

## Abstract

Aims: Considering the worldwide trend of an increased lifetime, geriatric trauma is moving into focus. Trauma is a leading cause of hospitalization, leading to disability and mortality. The purpose of this study was to compare the global health-related quality of life (HRQoL) of geriatric patients with adult patients after major trauma. Methods: This multicenter prospective registry-based observational study compares HRQoL of patients aged ≥65 years who sustained major trauma (Injury Severity Score (ISS) ≥ 16) with patients <65 years of age within the trauma registry of the German Trauma Society (DGU). The global HRQoL was measured at 6, 12, and 24 months post trauma using the EQ-5D-3L score. Results: We identified 405 patients meeting the inclusion criteria with a mean ISS of 25.6. Even though the geriatric patients group (≥65 years, *n* = 77) had a lower ISS (m = 24, SD = 8) than patients aged <65 years (*n* = 328), they reported more difficulties in each EQ dimension compared to patients <65 years. Contrary to patients < 65, the EQ-5D Index of the geriatric patients did not improve at 12 and 24 months after trauma. Conclusions: We found a limited HRQoL in both groups after major trauma. The group of patients ≥65 showed no improvement in HRQoL from 6 to 24 months after trauma.

## 1. Introduction

Since the geriatric population is forecasted to further increase, tremendous challenges are coming our way. The number of persons over 65 years of age in industrial societies is already over 20% and will reach over 35% by 2060 [1]. It is likely that the number of severely injured geriatric patients will rise in line with demographic trends. In 2017, a total of 26% of the patients included in the Trauma Register DGU^®^ of the German Trauma Society (DGU) were over 70 years old [2]. Physiological changes and comorbidities of the elderly population are challenging factors in treatment. The likelihood of severe impact on the quality of life even after minor injury increases with age, leading to hospitalization and impairment [3]. A generally decreased ability to tolerate stress imposed by traumatic incidents leads to a significant increase in social costs and risk of mortality [4,5].

In general, major trauma research applies mortality for the measurement of initial trauma severity and outcome [6]. Using patient-centered analysis methods like the multidimensional EQ-5D (European Quality of Life 5 Dimensions) score requires a more demanding longitudinal follow up [7]. However, the personal consequences of major trauma are generally severe, which makes investigating health-related quality of life (HRQoL) fundamentally necessary, especially in the vulnerable elderly patient population.

The purpose of this study is to compare the progress of HRQoL in patients equal or over 65 years of age to patients under 65 years of age after major trauma. We hypothesized that there is an impairment in evolution of HRQoL after major trauma in the elderly.

## 2. Methods

In a multicenter prospective registry-based observational cohort study within the East Bavarian trauma network (TNO), we monitored the development of health-related quality of life of adult patients after major trauma (ISS ≥ 16). The TNO is part of the trauma registry of the German Trauma Society (DGU), and consists of 25 hospitals. Two hospitals are maximum care facilities, 15 hospitals provide basic care and 8 hospitals provide standard care [8]. We assured excellent quality of the recorded data. As described in the methodical paper of this study by Koller et al. [9], substantial effort has been made, to exclude confounding factors and bias. The study was approved by the Ethics Committee of the University of Regensburg (reference number 10-101-0077). The study was funded by a grant from the German Federal Ministry of Education and Research (reference number 01GY1153), and was registered in the database of the German Network of Health Services Research (reference number VfD_Polyqualy_12_001978), as well as in the German Clinical Trials Register (reference number DRKS00010039).

**Figure 1:**
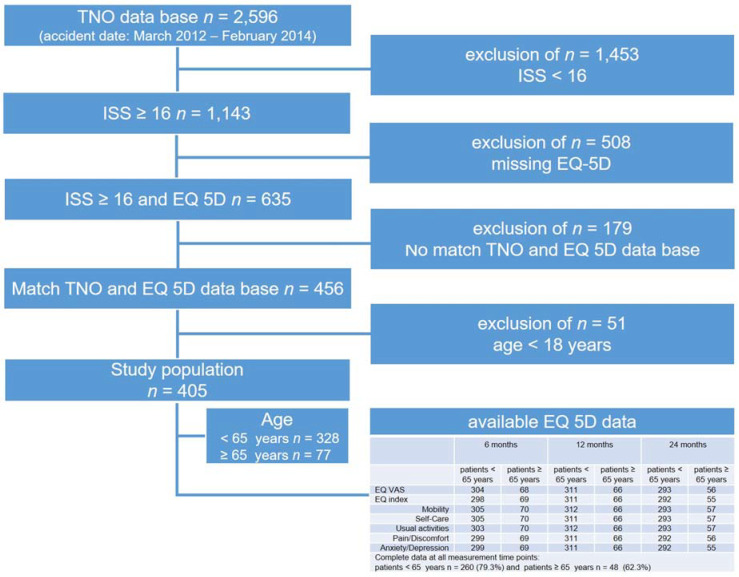
Selection procedure of the study population.

Between March 2012 and February 2014, 2596 patients were treated for severe trauma and were documented in the TNO data base. Overall, 56% of these patients (*n* = 1453 patients) had to be excluded due to an Injury Severity Score (ISS) below 16. EQ 5D data were collected from 508 patients between September 2012 and February 2016. For 456 patients, a match in both databases (TNO and EQ 5D) was complete. For the present analyses, patients below the age of 18 years were excluded, resulting in 405 patients with an age ≥ 18 years, ISS ≥ 16, and a HRQoL assessment (Figure 1). For the maintenance of a representative sample and the evaluation of bias, we compared the baseline data of included patients and patients lost to follow up. We found no clinically significant differences between participants and the excluded patient population. 

We used the web-based registry of the German Trauma Society (DGU) for data acquisition. It contains 130 single items per patient. For the present study, the following baseline data were used: sociodemographic data including age and sex, clinical data including level of care of the trauma center, mechanism of injury, intensive care, intubation and duration of intubation, ISS, Glasgow Coma Scale (GCS), ASA physical status (American Society of Anesthesiologists Score), Revised Injury Severity Classification II (RISC II) and Functional Capacity Index (FCI). 

Additionally, we assessed the global HRQoL, which was measured using the EQ-5D-3L at 6, 12, and 24 months after trauma. The first part of the EQ-5D evaluates the health status regarding mobility, self-care, usual activities, pain/discomfort, and anxiety/depression. The responses were dichotomized into no problems versus problems. Additionally, five dimensions were converted into EQ index ranging from −0.21 (worst health) to 1.00 (best health) [10]. The second part of the EQ-5D-3L measures the patient’s assessment of their current global health status and contains a 100 mm global health visual analog scale (EQ VAS) ranging from 0 points (worst health) to 100 points (best health) concerning the patient’s assessment of their current global health status. The EQ-5D is available in German and validated [11].

The ISS assess the severity trauma based on the Abbreviated Injury Scale (AIS). Each injury of the three most affected body regions is rated. The square of each score is then summed up to an ISS. A major trauma is defined as an ISS ≥ 16 [12].

The RISC II is a prognostic score evaluating different factors at initial evaluation of trauma patients. It comprises ten items of clinical and laboratory parameters to evaluate early mortality of the patient [13].

The FCI is a score used to estimate the patient’s level of functional impairment within the following year after trauma. It is based on ten physical functions which are evaluated and transformed into a numerical score on a scale of 0–100. An index of 100 represents no functional limitation [14].

## 3. Statistics

Statistical analysis was performed using the software package SPSS (Version 25, SPSS Inc, Chicago, IL, USA). The level of significance was set at *p* ≤ 0.05 for all tests. Data analyses were of an exploratory manner. Thus, no adjustments for multiple testing were conducted. Missing values were not imputed. The study was conducted in the context of health services research. No sample size calculation was conducted, as all patients were treated for major trauma between March 2012 and February 2014 with EQ 5D data, and at least at one of the three measurement time points were included.

Descriptive analyses were done using frequency (*n*), percentage (%), mean (m), standard deviation (sd), 95% confidence interval (95% CI), median (med), and interquartile range (IQR). Chi-square tests were used for categorial data and U-tests were used for metric data, in order to compare baseline characteristics of geriatric patients (≥65 years) and patients under the age of 65 years. Fisher-exact tests were used to compare five EQ dimensions (no problem versus any problem) between both patient groups.

Linear mixed models (maximum likelihood method, unstructured repeated covariance type) were used to evaluate repeated measures of HRQoL (i.e., EQ index and EQ VAS) 6, 12, and 24 months after trauma within and between patient groups (interaction effect time * patient group). Additionally, covariates RISC II (main effect) and FCI (main effect) and AIS for the body regions head, face, thorax, abdomen, extremities, and soft tissue (main effects) were included in the linear mixed linear models to adjust for injury severity. The mixed linear models use the full data set, and thus will give unbiased estimates.

## 4. Results

### 4.1. Baseline Characteristics

Four hundred and five patients met the inclusion criteria (Figure 1). There were 328 (81%) patients under 65 years of age and 77 (19%) patients aged 65 years or older. Overall, 72% of the patients were male. The majority (57%) of the patients were treated in a level II trauma center. Most patients sustained a high-velocity trauma in a traffic accident (32%). Most patients had to be treated in the intensive care unit (91%) and 52% of the patients had to be intubated. The mean time of intubation was 9 days (sd = 10). The mean ISS was 26 (sd = 10), the mean RISC II was 7 (sd = 15), and the mean FCI was 4 (sd = 1). Table 1 presents the baseline characteristics for the total sample and separately for geriatric patients and for patients under the age of 65 years and the mechanism of injury. 

### 4.2. Quality of Life

**Figure 2:**
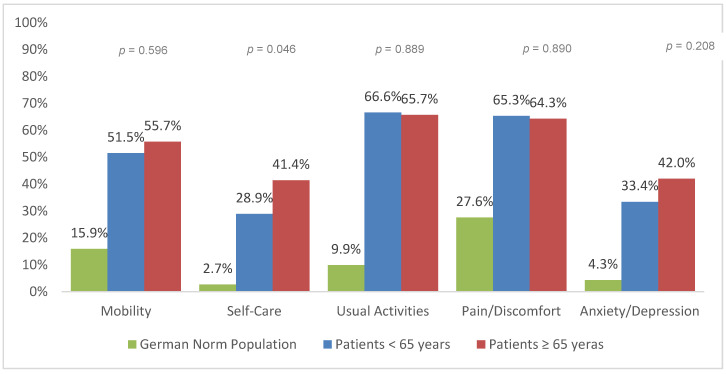
Reported problems in EQ-5D-3L dimension 6 months after trauma (European Quality of Life 5 Dimensions 3 Level Version).

**Figure 3:**
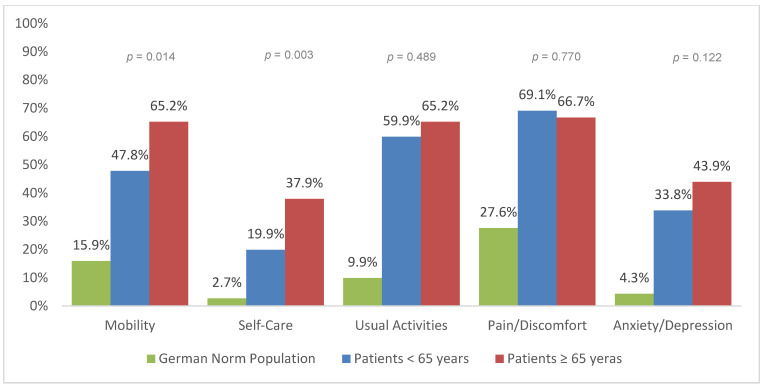
Reported problems in EQ-5D-3L dimensions 12 months after trauma (European Quality of Life 5 Dimensions 3 Level Version).

**Figure 4:**
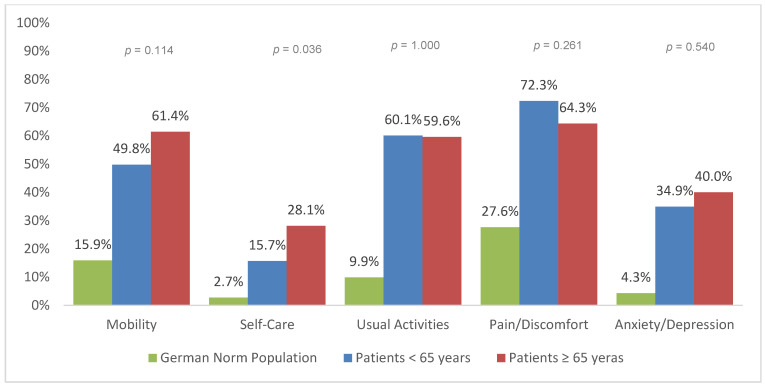
Reported problems in EQ-5D-3L dimension 24 months after trauma (European Quality of Life 5 Dimensions 3 Level Version).

The EQ-5D dimensions at 6, 12, and 24 months after trauma are presented for geriatric patients (≥65 years) and patients under 65 years of age (Figure 2, Figure 3 and Figure 4). In general, severe trauma patients reported more difficulties in all five HRQoL dimensions than the German general population. Geriatric patients reported significantly more impairment than patients under the age of 65 years of age. Significant differences between geriatric patients (≥65 years) and patients under 65 years of age were found for self-care at 6 months (*p* = 0.046), 12 months (*p* = 0.003), and 24 months post-trauma (*p* = 0.036). Significant differences between age groups were also found in mobility 12 months after trauma (*p* = 0.014).

The results of the mixed linear models support the notion that FCI, RISC II, AIS face, as well as AIS extremities, and ASA physical status have an impact on quality of life (Table 2). The EQ index and EQ VAS decreased with increasing RISC II as well as increasing ASA physical status, and increased with increasing FCI. With increasing AIS extremities, the EQ index and EQ VAS decreased.

Table 2 presents results of the main effects of AIS indices on quality of life.

**Figure 5:**
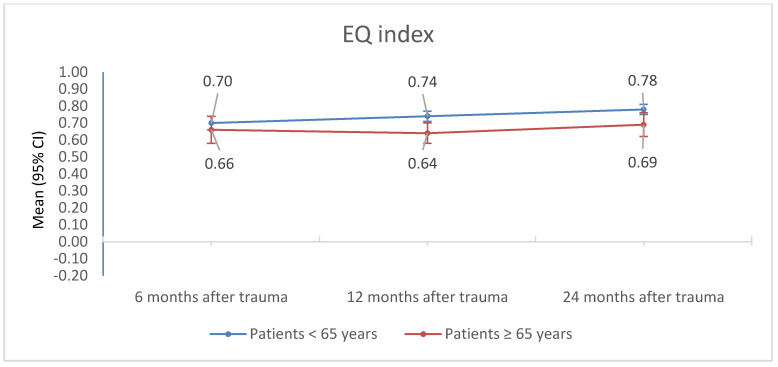
EQ index, adjusted for RISC II and FCI, of patients <65 years and ≥65 years of age 6, 12, and 24 months after trauma.

**Figure 6:**
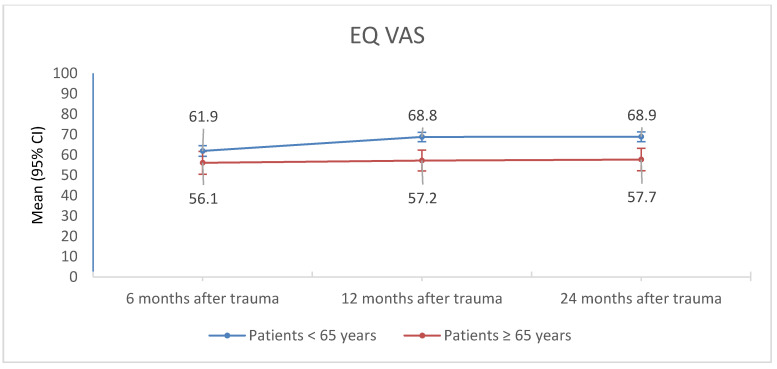
EQ VAS of patients <65 years and ≥65 years of age 6, 12, and 24 months after trauma.

Geriatric patients (≥65 years) reported a lower EQ index and EQ VAS 12 months and 24 months after trauma than patients under the age of 65 years (*p*-values < 0.012, (Table 3, Figure 5 and Figure 6). The EQ index as well as the EQ VAS of geriatric patients (≥65 years) did not significantly change from 6 to 24 months after trauma (Table 3, Figure 5 and Figure 6). The EQ index as well as the EQ VAS of patients under 65 years of age increased significantly from 6 to 12 to 24 months after trauma (Table 3, Figure 5 and Figure 6). Post hoc tests showed that the EQ index of patients under the age of 65 years increased significantly from 6 to 12 months post trauma (*p* = 0.037), and from 12 to 24 months post trauma (*p* = 0.028). Moreover, EQ VAS was significantly lower 6 months post trauma than 12 as well as 24 months post trauma (*p* values < 0.001), but did not differ between 12 and 24 months post trauma (*p* = 0.664).

The EQ index after 6, 12, and 24 months did not differ between male and female patients (Table 4). Moreover, the EQ index did not change over time within female and male patients (Table 4). Within male patients, no changes in EQ VAS from 6 to 24 months after trauma were found (Table 4). The EQ VAS of female patients increased significantly from 6 to 12 to 24 months after trauma (Table 4). Post hoc tests showed that EQ VAS was significantly lower 6 months post trauma than 12 as well as 24 months post trauma (*p* values < 0.001), but did not differ between 12 and 24 months post trauma (*p* = 0.736). However, female patients started with significantly lower EQ VAS than male patients (*p* = 0.036).

The EQ index as well as the EQ VAS of geriatric patients (≥65 years) did not significantly change from 6 to 24 months after trauma (Table 3, Figure 5 and Figure 6 ). The EQ index as well as the EQ VAS of patients under 65 years of age increased significantly from 6 to 12 to 24 months after trauma (Table 3, Figure 5 and Figure 6). Post hoc tests showed that the EQ index of patients under the age of 65 years increased significantly from 6 to 12 months post trauma, and from 12 to 24 months post trauma. 

Moreover, EQ VAS was significantly lower 6 months post trauma than 12 as well as 24 months post trauma, but did not differ between 12 and 24 months post trauma.

## 5. Discussion

To our knowledge, this is the first study comparing HRQoL after major trauma in different age groups with a focus on geriatric patients. We found a limited HRQoL in geriatric patients up to 24 months after major trauma. Contrary to patients under the age of 65 years, geriatric patients showed no significant improvement in HRQoL (EQ index and EQ VAS) from 6 to 24 months after trauma. 

The influence of age on mortality in severe injuries is a topic of ongoing research. Demetriades et al. found that geriatric trauma patients have a higher mortality in general, compared to younger patients, even after minor injury. Concordant with our results, they found that geriatric patients are also more likely to need intensive care unit (ICU) treatment and operations [15]. Kühne et al. and Morris et al. found that mortality rises with increasing age, regardless of ISS [16]. This is concordant with the findings in our study: The elderly had a lower mean ISS but a greater impairment in HRQoL. Grossman found that elderly trauma patients suffer more significant injuries compared to a younger cohort and have a higher mortality rate [17,18]. Sterling et al. found that death after same-level falls as a cause of death were 10 times more likely in the elderly [19]. The increased comorbidities in the elderly are often the reason for injury and contribute to worse outcome [4]. The unequal distribution of patients in the two groups in our study represents the typical age structure in major trauma. However, with a demographic trend towards the inverted population pyramid, a shift is to be expected. Rising numbers of elderly people will probably lead to an increase in geriatric trauma. Moreover, the baby boomers are starting to grow into the geriatric age group. It is assumed that this generation has a higher risk propensity [20] and a strong desire for optimum care and physical fitness and participation [21]. Therefore, this topic is gaining even more importance. 

As life expectancy increases, the probability of comorbidities such as osteoporosis, leading to complex injury patterns even after minor traumatic incidents, also increases [22,23]. A systematic review and meta-analysis of several articles by Sammy et al. investigated contributing reasons for mortality in geriatric trauma patients. The factors affecting mortality included age and gender, comorbidities and medication (especially blood-thinning medication), as well as injury type and severity. Interestingly, mortality after low-level falls in the elderly was shown to be higher than after high velocity injuries [24,25,26,27]. In our study, low-level falls were the second most common reason for major trauma in geriatric patients. Falls are a common mechanism for geriatric trauma patients. Even though they present an overall lower ISS, they often sustain severe injuries to the head [28]. Unsteady gait, vision, hearing, and polypharmacy increase the risk of collapse, and anticoagulation medication increases the risk of hemorrhage [29]. Head, chest and proximal femur fractures are also more likely to occur.^17^ For the comparison of the evolution of HRQoL over time, we included FCI, RISC II and the AIS of the body regions into the mixed linear model (MLM) to reduce confounding factors. Overall, the MLM revealed a significant impact of AIS face and AIS extremities on HRQoL 

The subscale analysis of EQ index revealed larger impairments in geriatric patients for the categories self-care and mobility. An increase in mobility and self-care in the elderly leads to an improved physical and mental quality of life [30]. A lack of mobility is associated with reduced independence, decreased mental function, falls and consequently death [31,32]. Therefore, means of increasing elderly patient’s mobility and involvement in self-care should be the main goal of rehabilitation. 

Younger adults had lower values for pain/discomfort. Several studies indicated a higher pain threshold in the geriatric population [33]. However, older people are not simply less sensitive to pain. Sofaer et al. suggested that old people see pain as a normal part of aging, and therefore perceive pain as normal and inevitable [34]. Hence, older patients tend to report pain to a lesser extent and demand pain medication less frequently [35]. Nonetheless, pain leads to suffering and disability. This is why multimodal pain management is compulsory, especially in the elderly [36]. Mędrzycka-Dąbrowska et al. found that proper pain management can reduce mortality in geriatric patients [37]. Therefore, evidence-based guidelines for in-hospital treatment and rehabilitation are needed to standardize the care of geriatric trauma patients [38,39,40].

The paradoxical finding that geriatric patients with low impact trauma are at higher risk than patients who sustained higher velocity trauma should reflect on the triage of geriatric trauma patients [41]. The question remains whether established trauma severity measurement tools are able to reflect the patient’s condition and if this is associated with the prognosis. Currently, the ISS is the standard evaluation instrument in the initial management of major trauma [16]. An ISS over 16 indicates major trauma associated with significant mortality. In the present study, only patients with an ISS greater than 16, indicating major trauma, were included. Although many patients with a lower ISS might be facing a comparable impairment of HRQoL, we focused on the high-risk population of geriatric major trauma reflected by an ISS over 16. Even though the ISS is a very effective tool to assess the severity of the injury and predict initial mortality, it has shown weakness in the prediction of prognosis and inclusion of interfering factors, such as comorbidities [24,28]. Therefore, the development of an assessment tool specifically for geriatric trauma for initial triage and further treatment might be valuable. Under-triage of geriatric patients often begins during the pre-hospital assessment. A worse general health status and preexisting conditions complicate clinical examination and the initial grading of injury severity [42]. Advanced age is associated with transport to a lower level of trauma care [43]. We also recorded a trend to a lower level of care of the primary admission facility with advanced patient age. In the literature, initial under-triage is associated with increased mortality [43]. Unconscious age bias occurs in pre-hospital providers, as well as hospitals, and therefore one must maintain a high clinical suspicion for serious injuries, regardless of the mechanism of injury [42,44]. The triage to a high-level trauma center, especially one with a high number of geriatric cases, is linked to better outcomes for the elderly [44]. A team approach with interdisciplinary care involving geriatricians, social workers, and pharmacists supervised by surgeons improves the quality of trauma care to address comorbidities, geriatric syndromes, care planning and rehabilitation, medication and pain management right from the beginning of hospitalization may reduce mortality and improve functional outcome [38,42]. Post-discharge issues are as important as the initial management of severe injuries in the elderly population. Post-acute rehabilitation and the patients’ functionality have a relevant impact on outcome [29]. There are few studies focusing on geriatric severe trauma. Measurement of outcome in geriatric trauma is mostly limited to mortality [6].

Facing the socio-economic impact of trauma and a decreasing mortality due to preventive measures and patient-centered outcome tools are increasingly relevant. More than ever, the aspect of HRQoL after severe trauma gains importance [45,46]. The main focus of this study was the assessment of posttraumatic HRQoL in geriatric major trauma patients. HRQoL can be defined as “how well a person functions in their life and his or her perceived wellbeing in physical, mental, and social domains of health” [47]. We used the EQ-5D-3L for HRQoL assessment 6, 12, and 24 months post trauma. Even though HRQoL decreases throughout life [48], we found significant impairment of HRQoL in geriatric patients compared to the general population. 

Our study also reveals a significantly worse recovery over time for geriatric patients. The impairment of major trauma on geriatric patient’s HRQoL is greater than in patients younger than 65 years. Contrary to geriatric patients, the control group under 65 years experienced a significant improvement in HRQoL within the first year after trauma. Even though PROM tools are gaining importance, HRQoL is under-researched in elderly severe trauma patients. Our study shows that geriatric patients particularly reported a loss of mobility, self-care and an increase in anxiety and depression. Few data exist on recovery for the elderly trauma patients. Undesirable conditions such as delirium, posttraumatic stress disorder, and depression might have a significant impact and lead to persistence in limitations of HRQoL [38]. In the last couple of years, ortho-geriatric trauma centers have been founded aiming for an improvement in the quality of care of elderly after trauma. Since this initiative was started after the closure of recruitment for this study, the question remains whether this has a significant impact on the quality of life after major trauma in the elderly.

The main limitation of our study is the limited number of cases. Even though major trauma in geriatrics is on the rise, there are only a limited number of cases even in a large trauma network like the TNO. Reporting on a specific patient cohort implicates a limitation of generalizability and bias. The low number of penetrating injuries is only one example, such that the data are specific for the reported patient population and the results might not be transferrable to other populations where the majority of patients sustain penetrating injuries due to violence. Another limitation is that due to the design of this large multicenter trial, an extension to clinical follow-up examinations was not possible. Therefore, we cannot present clinical follow-up data for correlation with HRQoL. Since this study was conducted in a heath-service context, there are many potential sources of bias or imprecision. Great efforts have been taken to improve data quality, as published before [9]. Assessment of data quality proved an excellent documentation rate of >95% in all major trauma patients. To reduce the impact of not uniformly distributed confounders between both age groups, the MLM included factors that were assumed to be potentially influencing the results, such as FCI, RISC II, and AIS of each body region. Furthermore, the MLM replaces missing values by using maximum likelihood estimates. Thus, all patients, even those with missing quality of life values at specific points of time, could be used for the analyses. 

In general, non-participation is a major issue in longitudinal studies. Our strategy for diminishing this effect was to compare baseline characteristics of participants with non-participants, which showed no significant discrepancy between the two groups. Still, participation dissent can introduce selection bias.

There might be other confounding factors that we did not take into account. However, including all major trauma patients in a specific geographic region and considering all clinically relevant factors, we aimed for unbiased estimates of HRQoL means. The strength of this study lies in its prospective multicenter registry-based design and data quality. Even though the study lacks a sample size calculation, we think it serves its purpose as a reference point when calculating sample sizes for further studies. However, further studies on the evaluation of HRQoL in post-traumatic conditions of the elderly would be valuable to confirm these results.

## 6. Conclusions

Our study is the largest elaboration on geriatric HRQoL after major trauma. We found a limited HRQoL measured by EQ-5D-3L in geriatric patients after major trauma. HRQoL of geriatric patients stagnated at a low level from 6 to 24 months after trauma. Compared to patients under the age of 65 years and the normal values of the general population, this suggests a relevant impairment of HRQoL after major trauma. Further evolution of ortho-geriatric initiatives is necessary to improve the care of the elderly after major trauma. 

## Figures and Tables

**Table 1:** Baseline characteristics of the study population.

		Patients < 65 years *n* = 328		Patients ≥ 65 years *n* = 77		*p* Value
Age (in years)		43.0	(24.3/53.0)	73.0	(70.0/78.0)	<0.001
Sex						
	male	245	(74.7%)	45	(58.4%)	0.007
	female	83	(25.3%)	32	(41.6%)	
Level of Trauma Center Facility						
	Level I	131	(39.9%)	21	(27.3%)	0.001
	Level II	186	(56.7%)	46	(59.7%)	
	Level III	11	(3.4%)	10	(13.0%)	
ISS		22.0	(18.0/30.0)	20.0	(17.0/27.5)	0.018
	AIS head	2.0	(0/3.0)	2.0	(0/4.0)	0.513
	AIS face	0	(0/0)	0	(0/0)	0.251
	AIS thorax	3.0	(2.0/4.0)	3.0	(0/4.0)	0.937
	AIS abdomen	0	(0/2.00)	0	(0/2.0)	0.186
	AIS extremities	2.0	(0/3.0)	2.0	(0/2.5)	0.020
	AIS soft tissue	0	(0/1.0)	0	(0/1.0)	0.995
RISC II ^1^		1.2	(0.7/3.9)	6.3	(2.2/16.5)	<0.001
FCI ^2^		4.0	(2.0/5.0)	5.0	(3.0/5.0)	0.073
ASA physical status ^3^		1.0	(1.0/1.0)	2.0	(2.0/3.0)	<0.001
GCS ^4^		15.0	(12.0/15.0)	15.0	(13.8/15.0)	0.266
Type of injury						
	blunt	309	(96.3%)	73	(97.3%)	1.000
	penetrating	12	(3.7%)	2	(2.7%)	
In hospital stay (in days)		15.6	(9.8/24.5)	17.0	(11.1/26.9)	0.215
Emergency surgery						
	no	215	(70.0%)	55	(80.9%)	0.075
	yes	92	(30.0%)	13	(19.1%)	
Time between accident and emergency surgery (in hours) ^5^		3.0	(2.4/4.5)	3.6	(1.9/6.4)	0.599
**stabbing**		33.8%		27.0%		
		22.8%		4.1%		
		6.5%		9.5%		
		4.0%		10.8%		
		16%		13.5%		
		8.6%		20.3%		
		1.2%		0.0%		
		3.2%		5.4%		
		0.6%		0.0%		

Data show median (IQR) for metric variables or number of patients (%, column percentage of all patients without missing values) for categorical variables; Level of Trauma Center Facility; Injury Severity Score (ISS); Revised Injury Severity Classification Score II (RISC II); Functional Capacity Index (FCI); ASA physical status (American Society of Anesthesiologists Score); Glasgow Coma Scale (GCS); *p*-value (comparison of patient age groups): U-test or Chi-squared test. ^1^ *n* = 300/*n* = 68; ^2^ *n* = 327/*n* = 77; ^3^ *n* = 309/*n* = 70; ^4^ *n* = 292/*n* = 62; ^5^ *n* = 85/*n* = 12.

**Table 2:** Impact of injury severity on quality of life.

		Estimate	95% CI		*p* Value
EQ index					
	FCI	0.04	0.02	0.06	<0.001
	RISC II	−0.003	−0.005	−0.001	0.001
	AIS head	−0.01	−0.02	0.01	0.315
	AIS face	0.03	0.00	0.06	0.026
	AIS thorax	−0.01	−0.03	0.01	0.198
	AIS abdomen	0.01	−0.01	0.03	0.178
	AIS extremities	−0.02	−0.04	0.00	0.016
	AIS soft tissue	−0.01	−0.04	0.03	0.742
	ASA physical status	−0.08	−0.12	−0.03	<0.001
EQ VAS					
	FCI	2.50	0.98	4.02	0.001
	RISC II	−0.23	−0.38	−0.09	0.001
	AIS head	−0.25	−1.60	1.09	0.712
	AIS face	1.86	−0.46	4.18	0.116
	AIS thorax	0.18	−1.16	1.52	0.789
	AIS abdomen	1.20	−0.24	2.64	0.101
	AIS extremities	−2.01	−3.49	−0.53	0.008
	AIS soft tissue	0.26	−2.41	2.93	0.847
	ASA physical status	−7.20	−10.63	−3.77	<0.001

Mixed linear models were used to assess the course of quality of life between patients aged <65 years and aged ≥65 years. These models were adjusted for injury severity measured by RISC II, FCI, AIS for each body region, and ASA physical status. Estimates of these injury severity parameters are presented.

**Table 3:** Course of quality of life data depending on age.

	6 Months Post Trauma				12 Months Post Trauma				24 Months Post Trauma				*p* Value ^2^
	*n*	m	95% CI		*n*	m	95% CI		*n*	m	95% CI		
**EQ Index**													
<65 years	256	0.70	0.66	0.74	268	0.74	0.70	0.77	247	0.77	0.74	0.81	0.004
≥65 years	55	0.69	0.61	0.77	55	0.69	0.61	0.76	44	0.73	0.64	0.81	0.558
*p* value ^1^		0.755				0.232				0.304			
**EQ VAS**													
<65 years	262	61.2	58.2	64.2	268	69.3	66.6	72.1	248	68.8	65.9	71.6	<0.001
≥65 years	55	59.4	53.2	65.6	55	62.7	57.0	68.5	45	63.8	57.3	70.3	0.262
*p* value ^1^		0.618				0.051				0.177			

Differences in quality of life between age groups and the course of quality of life within each age group were assessed by mixed linear models. Presented are the means (95% CI) of EQ index and global health visual analog scale (EQ VAS) for patients aged <65 years aged ≥65 years 6, 12, and 24 months after trauma adjusted for RISC II, FCI, AIS for six body areas, ASA physical status, and sex. AIS, Abbreviated Injury Scale; RISC II, Revised Injury Severity Classification Score II; FCI, Functional Capacity Index; ASA physical status, American Society of Anesthesiologists Score; ^1^ *p*-value of differences between age groups; ^2^ *p*-value of course of quality of life over time with each age group.

**Table 4:** Course of quality of life data depending on sex.

	6 Months Post Trauma				12 Months Post Trauma				24 Months Post Trauma				*p* Value ^2^
	*n*	m	95% CI		*n*	m	95% CI		*n*	m	95% CI		
**EQ Index**													
male	220	0.72	0.67	0.78	232	0.72	0.68	0.77	212	0.76	0.72	0.81	0.193
female	91	0.67	0.60	0.74	91	0.70	0.64	0.76	79	0.74	0.67	0.81	0.242
*p* value ^1^		0.209				0.546				0.548			
**EQ VAS**													
male	226	63.8	59.8	67.8	232	65.8	62.1	69.5	213	65.4	61.6	69.3	0.413
female	91	56.8	51.6	62.1	91	66.3	61.4	71.1	80	67.1	61.5	72.7	<0.001
*p* value ^1^		0.036				0.883				0.614			

Differences in quality of life between male and female patients and the course of quality of life within each sex were assessed by mixed linear models. Presented are the means (95% CI) of EQ index and EQ VAS for male and female patients 6, 12, and 24 months after trauma adjusted for RISC II, FCI, and AIS for six body areas, ASA physical status, and age. AIS, Abbreviated Injury Scale; RISC II, Revised Injury Severity Classification Score II; FCI, Functional Capacity Index; ASA physical status, American Society of Anesthesiologists Score; ^1^ *p*-value of differences between age groups; ^2^ *p*-value of course of quality of life over time with each age group.
